# An investigation of strong edge geodesic number on *m*-polar fuzzy environment and its application

**DOI:** 10.1371/journal.pone.0327882

**Published:** 2025-10-17

**Authors:** Tanmoy Mahapatra, Ebenezer Bonyah, Madhumangal Pal

**Affiliations:** 1 Department of Mathematics, Ramkrishna Mahato Government Engineering College, Purulia, West Bengal, India; 2 Department of Mathematics Education, Akenten Appiah Menka University of Skills Training and Entrepreneurial Development, Kumasi, Ghana; 3 Department of Applied Mathematics, Vidyasagar University, Midnapore, West Bengal, India; 4 Department of Mathematics and Innovation, Saveetha School of Engineering, Saveetha Institute of Medical and Technical Sciences, Chennai, Tamilnadu, India; Universidad de Cadiz, SPAIN

## Abstract

For crisp graphs, the notion of edge geodesic numbers has been known for a long time. But lately, the focus has shifted to investigating this idea in fuzzy graphs, which has resulted in studies of a number of properties. Determining a strong edge geodesic number in the context of a *m* -polar fuzzy graph (*m* PFG), where nodes and edges both have *m* membership values, poses special difficulties that call for creative solutions.

Strong geodesic numbers and strong edge geodesic numbers in *m* PFGs are defined in this study along with a detailed description. It determines an upper bound for strong edge geodesic numbers in a variety of well-known *m* PFGs. The metric space on the set of all vertices in a graph is associated with the strong edge geodesic distance. This article also discusses the sufficient and required conditions for robust edge geodesic cover. Additionally, isomorphic properties on strong geodesic distance are examined. Along with its various characteristics, the neighborhood notion on strong geodesic distance is also presented. Interesting characteristics of the latter are explored, and the connections between strong geodesic and strong edge geodesic numbers are analyzed. Additionally, the usefulness of strong edge geodesic numbers in *m* PFGs is illustrated via a practical application.

This work expands the scope of fuzzy graph theory and its applications by defining and analyzing these new notions, which offer deeper insights into the structural and dynamic features of *m* PFGs.

## 1 Introduction

### 1.1 Research background and related works

In various real-world scenarios, we often describe complex systems using models composed of nodes and connecting lines that represent specific relationships between pairs of nodes. Graph theory enables us to illustrate the existing connections within networks or systems. The application of graph theory has become standard practice across different contexts, including computer networks, electric grids, and more.

Some investigates the modeling and analysis of transportation and telecommunications networks using fuzzy graph theory. Due to various factors like traffic congestion, varied communication quality, or insufficient data, transportation and communication networks frequently deal with uncertainties and imprecise information. By introducing fuzzy sets into graph theory, where edges and vertices are given membership values to indicate varying degrees of connection or dependability, fuzzy graphs offer a useful framework for addressing these issues. The goal of this research is to employ fuzzy graphs to create models for the best possible path selection, network dependability, and efficiency in transportation and telecommunication systems. The outcomes show how fuzzy graph approaches may manage the complexity of the real world and provide better answers for network analysis and uncertain decision-making.

In modern settings, analysts recognize that the data that supports our perception of the world is rife with uncertainty. This uncertainty has a big impact on a lot of different areas of science and technology. Although the fuzzy set structure provides a way to express this uncertainty using membership values that range from 0 to 1, there are some situations in which conventional numerical representations are insufficient. Consider the challenge of determining the minimal number of traffic inspectors needed to effectively monitor an urban road network in the presence of multipolar fuzzy information. These situations are outside the purview of traditional fuzzy set theory and are closely related to the idea of a strong edge geodesic foundation. Therefore, it becomes necessary to present new approaches that can handle these complex, unpredictable datasets. It is this necessity that motivates the notion of a strong edge geodesic number in the context of *m*-polar fuzzy graphs. This conceptual breakthrough offers a sophisticated toolkit designed to handle the intricacies present in unpredictable multipolar information settings.

Zadeh [38] identified doubtfulness as a phenomenon and the ambiguity of real-life events in 1965. He presented a fuzzy set that altered people’s perceptions of science and technology. Fuzzy graphs were first proposed by Kafmann [18] using Zadeh’s fuzzy relation. Later, Rosenfeld [33] introduced the possibility of adding nodes and edges, in addition to other theoretical notions related to fuzziness, such as routes, connectedness, cycles, etc. Fuzzy graphs are then the topic of a lengthy discussion [26]. Numerous definitions and practical applications have been examined in [36]. Mordeson and Nair [27] also discuss some new concepts related to fuzzy graphs. The concept of a geodesic number of crisp graphs for vertices was first proposed by Frank Harary et al. [12,13,16]. Suvarna and Sunitha [35] expanded on the concept of geodesic distance in the context of fuzziness, while Linda and Sunitha [19] used *μ*-distance. Geodetic numbers of graphs and digraphs were introduced by Chang [17]. On perfect geodesic fuzzy graphs, Rehmani and Sunitha conducted research [34]. Atici introduced the graph’s edge geodetic number [9]. Originally proposed by Chen et al. [14], the *m*-polar fuzzy graph (*m*PFG). Density on *m*PFG was originally introduced later by Ghorai and Pal [15]. Moreover, Adeel and Akram have deeded on *m*PFGs and line graphs [1]. Akram et al. [2] concentrated on a few edge properties specific to *m*PFG. They also carried out their work on more different types of fuzzy graphs [4–8,37]. Mahapatra and Pal [20] later introduced fuzzy coloring of *m*PFG. Mahapatra and associates looked at fuzzy fractional coloring on fuzzy graphs more recently [21].Later on, they carried out their work on some different types of its generalization [22,23,29,30]. Mandal et al. [25] researched *m*PFG’s strong arcs.

Authors contributation towards geodesic as well as their generalization given in Table 1.

**Table 1:** Authors contributation towards geodesic as well as their generalization.

Authors	Years	Contributions
Kauffman	1973	Introduction of FG
Rosenfeld	1975	Modification of the concept of FG given by Kauffman
Bhutani and Rosenfeld	2003	Introduction of Strong arcs
Bhutani and Rosenfeld	2004	Introduction of geodesic distance
Suvarna and Sunitha	2009	Introduction of geodesic number of FG
Rehmani and Sunitha	2020	Introduction of edge geodesic number of FG
Mahapatra and Pal	In this paper	Introduction of strong edge geodesic number of *m*PFG

### 1.2 Motivation of the work

This paper explores the application of fuzzy graph theory in modeling and analyzing transportation and telecommunication systems. Transportation and communication networks often face uncertainties and imprecise information due to varying conditions such as traffic congestion, fluctuating communication quality, or incomplete data. Fuzzy graphs provide an effective framework to address these uncertainties by incorporating fuzzy sets into graph theory, where edges and vertices are assigned membership values to represent degrees of connectivity or reliability. This study focuses on developing models for optimal path selection, network reliability, and efficiency in transportation and telecommunication systems using fuzzy graphs. The results demonstrate the ability of fuzzy graph techniques to handle real-world complexities, offering improved solutions for network analysis and decision-making under uncertainty.

In contemporary contexts, analysts acknowledge the pervasive presence of uncertainty within the data that underpins our understanding of the world. This uncertainty holds significant sway across various domains of science and technology. While the framework of fuzzy sets offers a means to represent this uncertainty through membership values ranging between 0 and 1, there are scenarios where traditional numerical representations fall short. For example, think about the difficulty of estimating the bare minimum of traffic inspectors required to efficiently monitor an urban road network when multipolar fuzzy information is present. Such scenarios, intimately tied to the notion of a robust edge geodesic foundation, extend beyond the scope of conventional fuzzy set theory. Consequently, there arises a necessity to introduce novel methodologies capable of grappling with these multifaceted, uncertain datasets. This imperative drives the emergence of the concept of a strong edge geodesic number within the framework of *m*-polar fuzzy graph structures. This conceptual innovation provides a nuanced toolset tailored to navigate the complexities inherent in multipolar information landscapes characterized by uncertainty.

### 1.3 Organization of the paper

The format of this paper is as follows: Several definitions that are helpful in this manuscript are described in Sect 2. The meanings of strong geodesic cover, strong geodesic basis, and strong geodesic number were covered in sect 3. We looked at a few of their intersecting properties in this section. We presented the strong geodesic number for the edge version in Sect 4. The link between the strong geodesic number and the strong edge geodesic number of *m*PFG, as well as the lower and upper bounds, are some of their features that have been discussed. Sects. 5 have solved a real-life application based on traffic inspectors needed to patrol the urban road network system using a strong edge geodesic number of *m*PFG. Finally, the conclusion is found in Sects. 8.

### 1.4 Notations and symbols

In this section, we introduce and define key notations essential for the development of the theories presented throughout the paper. The table below, Table 2, outlines these notations along with their respective meanings for clarity and reference.

**Table 2:** Aberration of some useful terms.

Full name	Aberration Form
Fuzzy graph	FG
Underlying crisp graph	UCG
membership value	MV
*m*-polar fuzzy set	*m*PFS
*m*-polar fuzzy graph	*m*PFG
strength of connectedness	SC

## 2 Preliminaries

Here, introduction of some basic definitions is provided.

Let G=(V,E) be a graph, where *V* (non-empty set) is called vertex set and *E* (empty or non-empty set) is called edge set. If no edge incident with a vertex, then the vertex is said to be isolated vertex, otherwise, it is said to be non-isolated vertex.

In this article $p_{i},p_{s}:[0,1{]}^{m}\to [0,1]$ indicates the *i*^*th*^, *s*^*th*^ content of the projection mapping, with i,s=1(1)m standing in for i,s=1,2,...,m.

**Definition 1.** *[*38*] A fuzzy set A on the universal set X is characterized by a mapping* m:X→[0,1]*, which is called the membership function. A fuzzy set is denoted by* A=(X,m).

**Definition 2.** *[*18*] A fuzzy graph is a triplet* G=(V,σ,μ) *with underlying crisp graph* $G^{*}=(V,E)$ *where* σ:V→[0,1] *is a fuzzy set in V and* $\mu :\tilde{V^{2}}\to [0,1]$ *is a fuzzy set in* $\tilde{V^{2}}$ *such that* μ(a,b)≤inf{σ(a),σ(b)}*, for all* $(a,b)\in \tilde{V^{2}}$ *and* μ(a,b)=0*, for all* $\left(a,b)\in (\tilde{V^{2}}-E\right)$*. Here σ and μ are the fuzzy vertex and fuzzy edge of G respectively.*

**Definition 3.** *[*36*] The fuzzy graph* $H=(V_{1},\sigma_{1},\mu_{1})$ *is said to be a fuzzy subgraph of* G=(V,σ,μ) *if* $V_{1}\subseteq V$, $\sigma_{1}(a)=\sigma (a)$ *for all* $a\in V_{1}$ *and* $\mu_{1}(a,b)=\mu (a,b)$ *for all* $(a,b)\in \tilde{V_{1}^{2}}$.

**Definition 4.** *[*14*] An mPFS A (or a [0,1]*${\;\;}^{\text{m}}-set$*) on X is a mapping* $A:X\to [0,1{]}^{m}$. *m*(*X*) *indicates the set of all m-polar fuzzy sets on X.*

**Definition 5.** *[*14*] Let A be an mPFS. Then height of A is indicated by h(A) and is defined by*


$$(\underset{x\in A}{\sup}p_{1}\circ A(x),\underset{x\in A}{\sup}p_{2}\circ A(x),\ldots ,\underset{x\in A}{\sup}p_{m}\circ A(x)).$$

**Definition 6.** *[*15*] An mPFG* G=(V,A,B) *with UCG* $G^{*}=(V,E)$, $A:V\to [0,1{]}^{m}$ *as well as* $B:\tilde{V\times V}\to [0,1{]}^{m}$ *represents an mPFS of V and* $\tilde{V\times V}$ *respectively and which obey the rule such that* ∀i=1,2,3,…,m, $p_{i}\circ B(a,c)\leq inf\{p_{i}\circ A(a),p_{i}\circ A(c)\}$ *for all* $(a,c)\in \tilde{V\times V}$ *as well as* B(a,c)=0 *for all* $\left(a,c)\in (\tilde{V\times V}-E\right)$. $p_{i}\circ A(a)$ *and* $p_{i}\circ B(a,c)$ *indicates i*^*th*^ *component of MV of node a and edge (a,c).*

**Definition 7.** *[*15*]* $H=(\tilde{V},\sigma ,\gamma )$ *is considered a complete mPFG if* $p_{s}\;\circ \;\gamma (x,w)=p_{s}\;\circ \;\sigma (x)\wedge p_{s}\;\circ \;\sigma (w)$ *holds for every* $x,w\in \tilde{V}$ *and* s=1(1)m.

**Definition 8.** *[*15*] The mPF strong graph* $H=(\tilde{V},\sigma ,\gamma )$ *is identified when*


$$p_{s}\;\circ \;\gamma (x,w)=p_{s}\;\circ \;\sigma (x)\wedge p_{s}\;\circ \;\sigma (w)$$

*For each* $(x,w)\in \tilde{E}$ *and* s=1(1)m*, holds.*

**Definition 9.** *[*15*] Let* $H_{1}=(\tilde{V_{1}},\sigma_{1},\gamma_{1})$ *and* $H_{2}=(\tilde{V_{2}},\sigma_{2},\gamma_{2})$ *be two mPFGs of UCGs* $H_{1}^{*}=(\tilde{V_{1}},\tilde{E_{1}})$ *and* $H_{2}^{*}=(\tilde{V_{2}},\tilde{E_{2}})$ *respectively. A homomorphism from H*_1_ *to H*_2_ *is denoted by a mapping* $f:\tilde{V_{1}}\to \tilde{V_{2}}$ *such that*


$$p_{s}\;\circ \;\sigma_{1}(t_{1})\leq p_{s}\;\circ \;\sigma_{2}(f(t_{1}))$$

*and*


$$p_{s}\;\circ \;\gamma_{1}(t_{1},v_{1})\leq p_{s}\;\circ \;\gamma_{2}(f(t_{1}),f(v_{1})),$$

*for all* $t_{1}\in \tilde{V_{1}}$, $(t_{1},v_{1})\in \tilde{E_{1}}$ *and* s=1(1)m.

**Definition 10.** *[*15*] A bijective homomorphism between H*_1_ *and H*_2_ *is represented as a mapping* $f:\tilde{V_{1}}\to \tilde{V_{2}}$ *with the property*


$$p_{s}\;\circ \;\sigma_{1}(t_{1})=p_{s}\;\circ \;\sigma_{2}(f(t_{1})),\;for\;all\;t_{1}\in \tilde{V_{1}}\;and\;s=1(1)m,$$

*is called a weak isomorphism or (weak) node -isomorphism.*

**Definition 11.** *[*15*] A bijective homomorphism between H*_1_ *and H*_2_ *is a mapping* $f:\tilde{V_{1}}\to \tilde{V_{2}}$ *with the property* $p_{s}\;\circ \;\gamma_{1}(t_{1},v_{1})=p_{s}\;\circ \;\gamma_{2}(f(t_{1}),f(v_{1}))$, *for all* $(t_{1},v_{1})\in \tilde{E_{1}}$ *and* s=1(1)m, *is called a co-weak isomorphism or (weak) line-isomorphism.*

If *f* from *H*_1_ to *H*_2_ is a (weak) line-isomorphism as well as a (weak) node-isomorphism, it is referred to as an isomorphism between *H*_1_ and *H*_2_.

**Definition 12.** *[*20*] When an edge of the mPFG is* (t,w),t,w∈V $\frac{1}{2}\{p_{s}\;\circ \;\sigma (t)\wedge p_{s}\;\circ \;\sigma (w)\}\leq p_{s}\;\circ \;\gamma (t,w),s=1(1)m$ *is* H=(V,σ,γ)*. It is regarded as independently strong. It’s known as an independently weak edge if it isn’t.*

**Definition 13.** *[*20*] The definition of the edge strength (t,w) is*


$$p_{s}\;\circ \;I(t,w)=\frac{p_{s}\;\circ \;\gamma (t,w)}{p_{s}\;\circ \;\sigma (t)\wedge p_{s}\;\circ \;\sigma (w)},$$

*for*   s=1(1)m.

**Definition 14.** *[*3*] Given a mPFG with UCG* $H^{*}=(V,E)$*, let* H=(V,σ,μ)*. Consider a subgraph* $P=(V^{\prime },\sigma^{\prime },\mu^{\prime })$ *with UCG* $P^{*}=(V^{\prime },E^{\prime })$ *belonging to H. If* $\left(supp(V^{\prime }),supp(E^{\prime })\right)$ *is a cycle and there isn’t a unique* $(x,y)\in E^{\prime }$ *(the edge set of P) such that* $p_{s}\;\circ \;\mu (x,z)=\inf\{p_{s}\;\circ \;\mu (a,c):(a,c)\in E^{\prime }\}$*, for* s=1(1)m*, then P is referred to as a mPF cycle.*

**Definition 15.** *[*24*] Let* $P:d_{1},d_{2},\ldots ,d_{k}$ *be a path in H, and let* H=(V,σ,μ) *be a mPFG. Next, we use S(P) to indicate the path P’s strength, which may be found as follows:* $S(P)=(\min_{1\leq i<j\leq k}p_{1}\;\circ \;\mu (d_{i},d_{j}),\min_{1\leq i<j\leq k}p_{2}\;\circ \;\mu (d_{i},d_{j}),\ldots ,\min_{1\leq i<j\leq k}p_{m}\;\circ \;\mu (d_{i},d_{j}))=(\mu_{1}^{n}(d_{i},d_{j}),\mu_{2}^{n}(d_{i},d_{j}),\ldots ,\mu_{m}^{n}(d_{i},d_{j}))$.

*The path between d*_1_ *and d*_*k*_ *has the following SC:* $CONN_{G}(d_{1},d_{k})=(p_{1}\;\circ \;\mu (d_{i},d_{j}){\;\;}^{\infty },p_{2}\;\circ \;\mu (d_{i},d_{j}){\;\;}^{\infty },\ldots ,p_{m}\;\circ \;\mu (d_{i},d_{j}){\;\;}^{\infty })$*, where* $\left(p_{s}\;\circ \;\mu (a,d){\;\;}^{\infty })=\max_{n\in N}(\mu_{i}^{n}(a,d)\right)$.

**Definition 16.** *[*25*] For every s* = 1 *to m, a graph* G=(V,σ,μ) *is referred to as a mPF tree if there is a spanning mPF subgraph* $H=(V,\sigma^{\prime },\mu^{\prime })$ *that is also a mPF tree.* $p_{s}\;\circ \;\mu^{\prime }(a,d)>p_{s}\;\circ \;CONN_{H}(a,d)$ *is implied in* $p_{s}\;\circ \;\mu^{\prime }(a,d)=0$.

**Definition 17.** *[*3*] Suppose* G=(V,σ,μ) *is an mPFG. Then mPF open-nbd of a node x is indicated as N(x) and defined as* N(x)={z∈V:(x,z)∈E}. *Then the closed-nbd of a node x is indicated as N[x] and given by* N[x]=N(x)∪{x}.

## 3 Strong geodesic number of *m*PFG

The concept of edge geodesic numbers is a critical parameter in graph theory, used to determine the minimum number of edges that intersect all shortest paths (geodesics) between pairs of vertices in a graph. This metric has valuable real-life applications, particularly in optimizing network designs, improving transportation systems, and enhancing communication networks. In logistics and urban planning, edge geodesic numbers help identify critical routes or connections that must be maintained to ensure efficient movement across a network. In computer networks, they assist in determining key edges for data flow optimization and fault tolerance. Similarly, in social networks and biological systems, understanding edge geodesics aids in analyzing influential connections that influence system behavior. By applying edge geodesic numbers, real-world networks can be made more resilient, cost-effective, and efficient, highlighting the importance of this graph-theoretic parameter in practical applications.

In this section, a strong geodesic number of *m*PFG by using strong geodesic distance has been established. This is different from geodesic distance as here we want to calculate the geodesic distance only on a strong path that is only on *α*-strong as well as *β*-strong edges. Some useful properties for strong geodesic numbers have been discussed here.

**Definition 18.** *Let* G=(V,σ,μ) *be an mPFG and a,d be two nodes of G. Let P be a strong path from a to d. If there does not exist any shorter strong path other than P in between a to d, then the path P is called geodesic and the length of P is said to be a strong geodesic distance of a and d and which is indicated by d*_*g*_(*a*,*d*).

**Theorem 1.** *In a mPFG, strong geodesic distance On the set of vertices,* G=(V,α,μ) *is a metric space.*

**Proof.** Let *e*, *t* be two nodes in *V*. Then the strong geodesic distance between two nodes *e*,*t* is indicated as *d*_*g*_(*e*,*t*). To prove a strong geodesic distance is a metric, we have to show that it satisfies four properties of metric space.

1. Let $P:e=u_{1},u_{2},\ldots ,u_{n}=t$ be the shortest strong path in between *e* and *t*. Then $p_{i}\;\circ \;\mu (u_{j},u_{j+1})>0$, for i=1(1)m and j=1,2,…,n−1. This shows that the length of *P* is non-null. Hence *d*_*g*_(*e*,*t*) > 0. Therefore, *d*_*g*_(*e*,*t*) > 0, for all e,t∈V.
2. *d*_*g*_(*e*,*t*) = 0 holds iff *e* = *t*. Hence, *d*_*g*_(*e*,*t*) = 0 holds iff *e*, *t*.
3. $d_{g}(e,t)=d_{g}(e,t)$ satisfied as the shortest strong path from *e* to *t* is the same as the shortest strong path from *t* to *e*. Therefore, $d_{g}(e,t)=d_{g}(t,e)$.
4. Let *e*, *t*, *c* be three nodes in *V*. We are to show that $d_{g}(e,c)\leq d_{g}(e,t)\;+\;d_{g}(t,c)$. If possible, let $d_{g}(e,c)>d_{g}(e,t)\;+\;d_{g}(t,c)$. Let *Q* be a strong *m*PF path from *e* to *c*. Let *Q*_1_ be a strong *m*PF path from *e* to *t* and *Q*_2_ be a strong *m*PF path from *t* to *c*. Then $Q_{1}\cup Q_{2}$ be a strong *m*PF path from *e* to *c* which has the least length from the path *Q*, a contradiction. Therefore, $d_{g}(e,c)\leq d_{g}(e,t)\;+\;d_{g}(t,c)$.

Therefore, a strong geodesic distance in an *m*PFG G=(V,α,μ) is a metric space.

**Definition 19.** *Let’s consider a connected mPFG denoted by* G=(V,α,μ)*. Suppose we have a subset of nodes* U⊆V*. We define the strong geodesic closure of U, denoted as (U), as the set comprising all nodes in U along with those nodes lying on strong geodesics connecting nodes within U. If* (U)=V*, we refer to U as a strong geodesic set or a strong geodesic cover. A strong geodesic basis is any minimal subset of nodes within a strong geodesic cover, and the cardinality of this basis is termed the strong geodesic number of G, indicated by gn(G).*

An example that demonstrates every definition above is given below.

**Fig 1:**
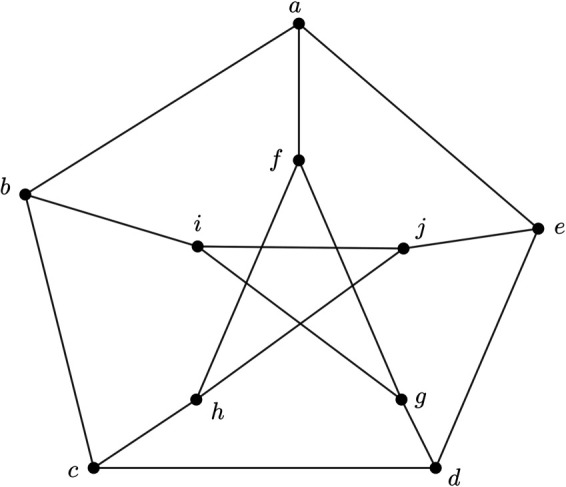
3PFG *G*.

**Example 1.** *Here, we examine a 3PFG illustrated in* Fig 1 *to illustrate the aforementioned definitions.*

*We give the MV of nodes as well as edges of G in tabular form in* Table 3 *and* 4.

**Table 3:** Vertex membership value.

Vertex	MV
a	(0.3,0.5,0.7)
b	(0.6,0.8,0.8)
c	(0.5,0.7,0.8)
d	(0.4,0.5,0.6)
e	(0.3,0.5,0.7)
f	(0.4,0.5,0.6)
i	(0.5,0.7,0.8)
j	(0.6,0.8,0.8)
g	(0.3,0.5,0.7)
h	(0.6,0.8,0.8)

**Table 4:** Edge membership value.

Edge	MV
(a,b)	(0.3,0.4,0.6)
(b,c)	(0.2,0.3,0.4)
(c,d)	(0.3,0.4,0.6)
(d,e)	(0.2,0.3,0.4)
(e,a)	(0.3,0.4,0.6)
(f,a)	(0.2,0.3,0.4)
(f,h)	(0.3,0.4,0.6)
(f,g)	(0.2,0.3,0.4)
(b,i)	(0.3,0.4,0.6)
(i,j)	(0.2,0.3,0.4)
(i,g)	(0.3,0.4,0.6)
(c,h)	(0.2,0.3,0.4)
(h,j)	(0.3,0.4,0.6)
(d,g)	(0.2,0.3,0.4)
(e,j)	(0.3,0.4,0.6)

*After some routine calculation, we can say that all the edges in G are either α-strong or β- strong. If we take* U={b,e,h,g}⊂V*, then we can easily see that U includes all the nodes in U and any other nodes that not include in U must lie on the strong geodesics connecting the nodes within U. Therefore, its fulfill the condition of definition 19. Hence,* (U)=V*. Therefore, U is a strong geodesic cover of G. It is evident that U is a minimally node-counting strong geodesic cover. Hence, U is a strong geodesic basis of G and a strong geodesic number is 4 that is,* gn(G)=4.

**Remark 1.** *If all the edges of an mPFG whose UCG is a Peterson graph are strong, then* gn(G)=4.

**Theorem 2.** *The strong geodesic number of a complete mPFG having a n number of nodes is n.*

Proof. Suppose *G* as a complete *m*PFG. Consequently, it is devoid of *δ*-strong arcs, implying that all edges within *G* are strong edges. Take two nodes, *x* and *y*, within *G*. As G^*^ is complete, there exists an edge connecting *x* and *y*, forming a strong geodesic (*x*,*y*). This strong geodesic doesn’t contain any arcs between other pairs of nodes within *G*, indicating that U=V serves as a strong geodesic cover for *G*. To establish it as a strong geodesic basis, we need to demonstrate that *U* has minimal cardinality. Suppose there exists another strong geodesic cover *T* of *G* with fewer nodes compared to *U*. In this case, at least one node in *U* is absent in *T*. Let’s denote this node as *a*. Consequently, the edges incident to *a* do not lie on any strong geodesic connecting pairs of nodes within *W*, hence violating the condition of *T* being a geodesic cover of *G*. Therefore, *U* stands as a geodesic basis of *G*. Hence, gn(G)=n.

**Theorem 3.** *The strong geodesic number is the same for two mPFG* $H,H^{\prime }$*, where* $H≅H^{\prime }$.

**Proof.** Suppose H=(V,σ,μ) as well as $H^{\prime }=(V^{\prime },\sigma^{\prime },\mu^{\prime })$ be two *m*PFGs. Since $H≅H^{\prime }$, then there exists a mapping $\phi :H\to H^{\prime }$ such that for each s=1(1)m

1. $p_{s}\;\circ \;\sigma (e)=p_{s}\;\circ \;\sigma^{\prime }(\phi (e))$, ∀ e∈V.
2. $p_{s}\;\circ \;\mu (e,t)=p_{s}\;\circ \;\mu^{\prime }(\phi (e),\phi (t))$, $\forall \;(e,t)\in \tilde{V^{2}}$.

Suppose *H* have a strong geodesic number *n*. Then there is a node-set S⊆V such that *S* contains *n* number of nodes and (S)=V. Suppose $S=\{a_{1},a_{2},\ldots ,a_{n}\}$. As $H≅H^{\prime }$, then the isomorphic image of the vertices *a*_*i*_, i=1,2,…,n is also cover the node set $V^{\prime }$. Let $S^{\prime }=\{\phi (a_{1}),\phi (a_{2}),\ldots ,\phi (a_{n})\}$. Then we can claim that $S^{\prime }$ is a strong geodesic basis of $H^{\prime }$. If possible, let $S^{\prime }$ is not a strong geodesic basis of $H^{\prime }$. Since *ϕ* is a bijective mapping between $H,H^{\prime }$, therefore there exists $\phi^{-1}:H^{\prime }\to H$ which is also a bijective mapping. Then we must have $\phi^{-1}(S^{\prime })=S$, which shows that *S* is not a strong geodesic basis of *H*, a contradiction. Hence, $S^{\prime }$ is a strong geodesic basis of $H^{\prime }$. Thus $gn(H^{\prime })=n$.

## 4 Strong edge geodesic number of *m*PFG

In this section, strong edge geodesic numbers by using strong geodesic distance for *m*PFG have been established. In previous discussions, we have seen that strong geodesic distance is different from ordinary geodesic distance. Therefore, it is totally a new concept which will be discussed thoroughly in this section. Some necessary and sufficient conditions for strong edge geodesic numbers have also been studied here.

**Definition 20.** *Let us consider a mPFG, represented by* H=(V,σ,μ)*, where the collection of nodes is indicated by V. Let* $H^{*}=(V,E)$ *represent the underlying crisp graph (UCG) of H. Let S represent a group of nodes in the mPFG. The strong geodesic closure of S, shown by* (*S*)_*e*_*, is the set of edges that lie on the strong geodesics linking the nodes in S. A set* (*S*)_*e*_ = *E denotes the strong edge geodesic cover of H if all of the edges in the mPFG are included.*

**Definition 21.** *The number of elements in a strong edge geodesic basis is known as the strong edge geodesic number, and it is denoted by gn*_*e*_(*H*)*. A strong edge geodesic cover with the lowest cardinality is referred to as a strong edge geodesic basis.*

The aforementioned definitions are exemplified through the following example provided below.

**Example 2.** *Here, we consider an 3PFG* H=(V,σ,μ) *having UCG* $H^{*}=(V,E)$ *to depict the above definitions.*

*If we consider* S={a,c}*, then we see that* (*S*)_*e*_ = {{*a*,*b*},{*b*,*c*},{*c*,*d*},{*a*,*d*}} = *E and* (S)={a,b,c,d}=V*, Therefore gn*_*e*_(*H*) = 2 *and* gn(H)=2*. Hence, we may say that gn*_*e*_(*H*) = *gn*(*H*)*, for some certain mPFG H. One simple observation is that if we consider S*_1_ = {*b*,*d*}*, then also* $(S_{1}{)}_{e}=\{\{a,b\},\{b,c\},\{c,d\},\{a,d\}\}=E$ *and* $(S_{1})=\{a,b,c,d\}=V$*. In this case, we also have gn*_*e*_(*H*) = 2 *and* gn(H)=2*. Here, we can also see that a strong edge geodesic basis is not unique.*

*Next, we consider another 3PFG* $H_{1}=(V_{1},\sigma ,\mu )$ *having UCG* $H_{1}^{*}=(V_{1},E_{1})$ *whose vertex set* $V_{1}=\{a,b,c,d\}$ *and having 5 edges* {(a,b),(a,c),(a,d),(b,c),(c,d)} *shown in* *Fig 3* *where we can not find any strong edge geodesic basis for H*_1_.

*Here, we see that no such set* $S\subset V_{1}$ *exists for which* $(S{)}_{e}=E_{1}$ *holds.*

**Fig 2:**
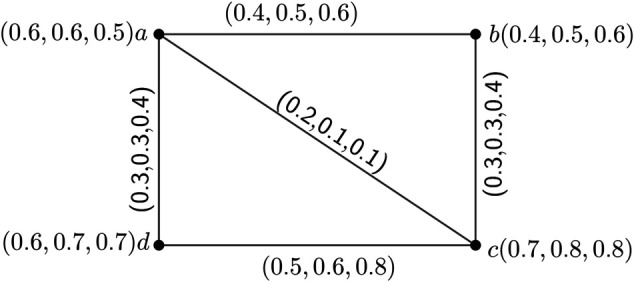
3PFG *H* having four nodes and five edges to illustrate the definition 19.

**Fig 3:**
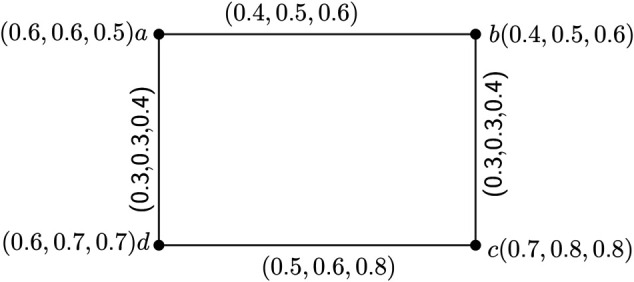
3PFG *H*_1_ having four node and five edges.

**Note 1.** *One simple observation from the* *Fig 2* *is that it has no δ-strong arcs and from the* *Fig 3* *we see that it has a δ-strong arcs, namely (a,c).*

Thus, the following theorem is established.

**Theorem 4.** *A connected mPFG* H=(V,σ,μ) *must have a strong edge geodesic cover iff it has no δ-strong arcs.*

**Proof.** Let us consider H=(V,σ,μ) have a strong edge geodesic cover. Suppose, *U* is a strong edge geodesic cover of *H*. We are to show that it has no *δ*-strong arcs. If possible, let us assume that it has a *δ*-strong arcs, namely (*x*,*y*). In light of the definition of a strong edge geodesic cover, we may therefore conclude that, in contrast to the assertion that *U* is a strong edge geodesic cover of *H*, the arc (*x*,*y*) does not contain in any strong edge geodesic path connecting the nodes of *U*. Therefore, there are no *δ*-strong arcs in *H*.

On the other hand, consider that *H* lacks any *δ*-strong arcs. As such, it only includes arcs that are *β* or *α*-strong. As a result, every arc in *H* is strong. As a result, a strong edge geodesic cover of *H* is formed by *V*.

**Theorem 5.** *The strong edge geodesic number is the same for two mPFG* $H,H^{\prime }$, *where* $H≅H^{\prime }$.

**Proof.** Suppose H=(V,σ,μ) as well as $H^{\prime }=(V^{\prime },\sigma^{\prime },\mu^{\prime })$ be two *m*PFGs. Since $H≅H^{\prime }$, then there exists a mapping $\phi :H\to H^{\prime }$ such that for each i=1(1)m.

1. $p_{i}\;\circ \;\sigma (e)=p_{i}\;\circ \;\sigma^{\prime }(\phi (e))$, ∀ e∈V.
2. $p_{i}\;\circ \;\mu (e,t)=p_{i}\;\circ \;\mu^{\prime }(\phi (e),\phi (t))$, $\forall \;(e,t)\in \tilde{V\times V}$.

Suppose *H* has a strong edge geodesic number *n*. Then there exists a node set U⊆V such that *U* contains *n* number of nodes and (U)=E. Suppose $U=\{a_{1},a_{2},\ldots ,a_{n}\}$. As $H≅H^{\prime }$, then the isomorphic image of the vertices *a*_*i*_, i=1,2,…,n is also cover the node set $V^{\prime }$. Let $U^{\prime }=\{\phi (a_{1}),\phi (a_{2}),\ldots ,\phi (a_{n})\}$. Then we can claim that $U^{\prime }$ is a strong edge geodesic basis of $H^{\prime }$. If possible, let $U^{\prime }$ is not a strong edge geodesic basis of $H^{\prime }$. Since *ϕ* is a bijective mapping between $H,H^{\prime }$, therefore there exists $\phi^{-1}:H^{\prime }\to H$ which is also a bijective mapping. Then we must have $\phi^{-1}(U^{\prime })=U$, which shows that *U* is not a strong edge geodesic basis of *H*, a contradiction. Hence, $U^{\prime }$ is a strong edge geodesic basis of $H^{\prime }$. Thus $gn_{e}(H^{\prime })=n$.

**Theorem 6.** *Given a connected mPFG* H=(V,σ,μ) *with no δ-strong arcs and a full mPFG K*_*n*_ *with n nodes, the strong edge geodesic number of H is n according to UCG* $H^{*}=K_{n}$.

**Proof.** Since there are no *δ*-strong arcs in *H*, all of its edges are strong edges. Assume that *H* has two nodes, *x*,*y*. Given the completion of H^*^, an edge connects *x*,*y*. The geodesic (*x*,*y*) is strong for the nodes *x*,*y*. In *H*, there isn’t an arc that is on the strong geodesic connecting any two nodes. Therefore, a strong edge geodesic cover of *H* is U=V. We must demonstrate that *U* has the least cardinality to establish that it is a strong edge geodesic basis. Assume that *W* is an additional strong edge geodesic cover of *H* that has fewer nodes than *U*. Afterwards, at least one node in *U* does not exist inside *W*. Let such a node be *a*. Hence, *W* is not an edge geodesic cover of *H* since the edges incident with *a* do not lie on any strong geodesic linking pairs of nodes in *W*. As a result, an edge geodesic basis of *H* is *U*. Hence, strong edge geodesic number which is denoted by *gn*_*e*_(*H*) as stated in definition 21 must be *n*.

**Proposition 1.** *For any complete mPFG* H=(V,σ,μ)*, the strong edge geodesic number is n, where* n=|V|.

**Proof.** This proposition clearly follows from the above theorem.

The converse part of the Theorem 6 as well as Proposition 1 may not always be true. This is depicted through an example.

**Example 3.** *Here, we consider a 3PFG H having four nodes whose UCG is K*_4_.

**Fig 4:**
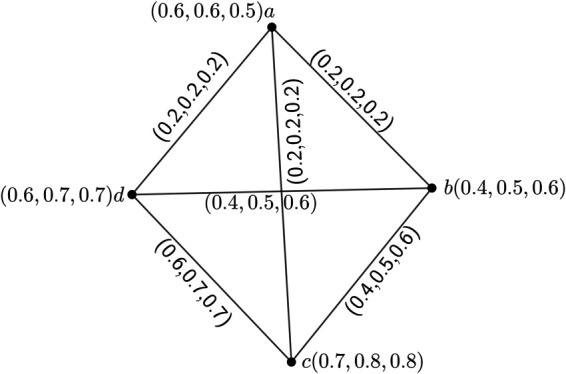
3PFG *H*.

*In* *Fig 4, we see that all the edges of H are strong. If we take* S={a,b,c,d}*, then* (S)=V *and S is a strong edge geodesic basis of H. Hence, gn*_*e*_(*H*) = 4 *but the given graph is not a complete 3PFG as for the edges (a,d) and (a,b),* $p_{i}\;\circ \;\mu (a,b)=\{p_{i}\;\circ \;\sigma (a)\wedge p_{i}\;\circ \;\sigma (b)\},\;i=1(1)m$ *not holds.*

**Remark 2.** *From the Theorem 2 and 6, we can conclude that for any complete mPFG H, we have gn*(*H*) = *gn*_*e*_(*H*).

Next, we attempt to determine the strong edge geodesic number of a linked *m*PFG, both its largest lower bound and its least upper bound.

**Theorem 7.** *For any connected mPFG* H=(V,σ,μ) *having no δ-arcs, we have* $2\leq gn_{e}(H)\leq n$*, where n is the number of nodes of H.*

**Proof.** Clearly, any strong edge geodesic cover must have at least two nodes. So $gn_{e}(H)\geq 2$.

As *H* is free from any *δ*-arcs, therefore the strong edge geodesic cover set can have at most *n* nodes, where *n* is the number of nodes of *H*. As, if we consider a complete *m*PFG then the strong edge geodesic cover set has at *n* nodes. In all other cases, the strong edge geodesic cover set can have at most *n* nodes. Hence, $gn_{e}(H)\leq n$.

Combining all the above cases, we have $2\leq gn_{e}(H)\leq n$.

**Theorem 8.** *For any complete bipartite mPFG* H=(V,σ,μ)*, where* $V=V_{1}\cup V_{2}$ *and* $|V_{1}|=p$, $|V_{2}|=q$*, gn*_*e*_(*H*) = *min*{*p*,*q*}.

**Proof.** Three scenarios will be examined in order to demonstrate this theory.

**Case 1.** *Let* $|V_{1}|=|V_{2}|=1$*. Then by using the Proposition 1, we can prove the theorem.*

**Case 2.** *Let* $|V_{1}|=1$ *and* $|V_{2}|\geq 2$*. This part can be proved by using the Theorem 6.*

**Case 3.** *Let* $|V_{1}|\geq 2$ *and* $|V_{2}|\geq 2$*. Without sacrificing generality, we can presume that p*<*q. Let* $V_{1}=\{a_{1},a_{2},\ldots ,a_{p}\}$ *and* $V_{2}=\{b_{1},b_{2},\ldots ,b_{q}\}$*. We have to prove that* $U=V_{1}$ *is an edge geodesic cover of H. Any edge* $\left(a_{i},b_{j}\right)$ *lies on the geodesic* $a_{i}-b_{j}-a_{k}$ *joining the nodes of U. Thus U is a strong edge geodesic cover of H.*

*To prove the theorem it only remains to show that U has the minimum cardinality. If possible, let S be another collection of nodes having less cardinality compared to U. Consider the following cases:*

**Case a:** *Let* $S\subset V_{1}$*. Then there exists at least one node say a*_*t*_ *in V*_1_ *such that* $a_{t}\notin S$*. For any edge* $\left(a_{t},b_{l}\right)$*, the only strong geodesic containing* $a_{t}-b_{l}-a_{s}$ *and* $b_{l}-a_{t}-b_{j}$*. Consequently, no strong geodesic connecting two nodes of S may include* $\left(a_{t},b_{l}\right)$*. As a result, S is not a geodesic cover of H with a strong edge.*

**Case b:** *Let* $S\subset V_{2}$*. Then in a similar way as stated above, It is demonstrable that S is not a geodesic cover with a strong edge on H.*

**Case c:** *Let* $S\subset V_{1}\cup V_{2}$*. This case only arises when S contains a node from V*_1_ *and a node from V*_2_*. As* |S|<|U|*, their exists a node* $a_{i}\in V_{1}$ *and a node* $b_{j}\in V_{2}$ *such that* $a_{i},b_{j}\notin S$*. At this point, the edge* $\left(a_{i},b_{j}\right)$ *does not connect any two nodes of S with a strong geodesic. Hence, S is not a strong edge geodesic cover of H.*

Thus, *U* is a strong edge geodesic basis of *H*. Hence *gn*_*e*_(*H*) = *p*. Thus, *gn*_*e*_(*H*) = *min*{*p*,*q*}.

Next, we find some relation between strong geodesic basis as well as strong edge geodesic basis of a connected *m*PFG. After finding their relation, we can easily find the least upper bound of the strong edge geodesic basis of any connected *m*PFG.

**Theorem 9.** *Suppose H is an mPFG. Each strong edge geodesic cover of H is also a strong geodesic cover of H.*

**Proof.** Let *U* be a geodesic cover of *H* with a strong edge. Assume that *x* is an incident node of *H* with respect to *y*. The edge (*x*,*y*) then rests on a strong geodesic linking to one or more nodes in *U*. Subsequently, it becomes apparent that *x* represents a node situated on a strong geodesic connecting a pair of nodes within *U*. *U* is a strong geodesic cover of *H* since *x* is arbitrary. Thus, the theorem.

**Theorem 10.** *Suppose H is an mPFG. Then* $gn(H)\leq gn_{e}(H)$.

**Proof.** Let *U* be a strong edge geodesic basis of *H*. Then, *gn*_*e*_(*H*) = |*U*|. Again, from the Theorem 9, we can say that *U* is a strong geodesic cover of *H*. Therefore, gn(H)≤|U|. Hence, $gn(H)\leq gn_{e}(H)$, for any *m*PFG *H*.

**Theorem 11.** *Suppose H is an mPF tree having no δ-strong arcs. Then H has a unique strong edge geodesic basis containing mPF end nodes.*

**Proof.** Assume that *U* is the set of all *m*PF end nodes in *H*. According to [24]’s Theorem 3.19, *H* lacks *β*-strong arcs because it is a *m*PF tree. Once again, *H* has no *δ*-strong arcs according to the criteria specified. As a result, *H* has *α*-strong arcs in every direction. Consequently, *H* is a *m*PF tree, and H* is a tree as well. H* and *H* have identical end nodes. Once more, we are aware that there is only one path connecting any two nodes in H*. Then each arc in *H* has to be a part of a strong geodesic connecting two nodes in *U*. Therefore, *U* is a geodesic cover of *H* with a strong edge.

It just has to be demonstrated that *U* has the lowest cardinality of all the strong edge geodesic covers of *H* to establish that *U* is a strong edge geodesic basis of *H*. Let *T* be an additional strong edge geodesic cover of *H*, if at all possible, with less cardinality than *U*. Then, one node in *U*, let’s call *a*, exists and is not a part of *T*. Given that H* is a tree, the edges that incident with *a* do not form a strong geodesic connecting any two nodes in *U*. This leads to a contradiction: *U* is not a strong edge geodesic cover of *H*. Thus, a strong edge geodesic basis of *H* is *U*.

**Corollary 1.** Let H be an mPFG where H* (the underlying crisp graph of H) is a tree. If H* *is a star graph, then gn*_*e*_(*H*) = *n* − 1*, where* |V|=n.

**Proof.** As H* (the underlying crisp graph of *H*) is a star graph, therefore we can think of it as *K*_1,*n*−1_ as it’s exactly one node connected with remaining with *n*–1 nodes. Since *H* is an *m*PF tree, therefore it is free from *δ*-strong arcs. Then by Theorem 11, we know that all the end nodes of *H* together form a unique strong edge geodesic basis of *H*. As *H* contains *n*–1 end nodes therefore *gn*_*e*_(*H*) = *n* − 1.

This corollary can be generalized for a special type of *m*PFG. The generalized version is given below as follows:

**Theorem 12.** *Suppose H be an mPFG which is free from δ-strong arcs and every vertex of H has MV*

(1,1,…,1)*. If there exists a vertex whose degree for each component is n–1, then* $gn_{e}(H)=n-1$.

**Proof.** Considering that *H* is a *m*PFG devoid of *δ*-strong arcs. We therefore have a unique strong edge geodesic cover of *H* according to the Theorem of 11. Let *a* represent the node whose degree is *n*–1 for every component. Assume U=V−{a}. We must first demonstrate that *U* is a geodesic cover with a strong edge of *H*. We have two cases for this.

**Case 1:** *a* edges the incidence.

Assume that the edge incident with *a* is (*a*,*c*). Given that *a* is the only degree node whose MV’s individual components are *n*–1, therefore *a* is incident with every vertex in *H* and there exists at least one node, say *d* such that *d* is a neighbour of *a* and *c*,*d* are not adjacent as *a* is the only node of degree whose each component of MV is *n*–1. Thus the edge (*a*,*c*) lies on the strong geodesic *d*–*a*–*c* joining the nodes of *U*. In similar manner we can show that *U* cover all the edges of *G*.

**Case 2:** Edges not incident with *a*.

Consider the edge (*e*,*f*) which is not incident with *a*. As e,f∈U, therefore the edge (*e*,*f*) lies on the strong geodesic connecting the nodes *e*,*f*. Thus *U* covers the edge (*e*,*f*). In the same way, we can prove that *U* covers all the edges of *H* which are not incident with *a*.

Combining both cases, we can conclude that *U* is a strong edge geodesic cover of *H*.

To show *gn*_*e*_(*H*) = *n* − 1, we have to show that *U* is a strong edge geodesic basis of *U* that is, we have to show that there does not exist another node set having cardinality *n*–2. If possible, let *S* be a strong edge geodesic cover of *G* having cardinality *n*–2. Let x,z≠S.

**Case a:** Let a∈S.

(*a*,*x*) or (*a*,*z*) does not lie on any strong geodesic connecting nodes of *S* since x,z≠S. Hence, *S* is not a geodesic cover of *H* with a strong edge.

**Case b:** a≠x and a≠z if a∉S. Then, *S* is not a strong edge geodesic cover of *H* since there is no geodesic joining the nodes of *S* via (*a*,*x*) or (*a*,*z*). From all of the cases, we can say that *U* is a strong edge geodesic basis of *H*. Thus *gn*_*e*_(*H*) = *n* − 1.

Next, we will focus on strong edge geodesic cover in light of the neighbourhood concept of an *m*PFG.

**Theorem 13.** *Consider a mPFG with no δ-strong arcs, denoted by* H=(V,σ,μ)*. For some* u∈N(v)*, let* v∈V *be such that* N(v)⊆N[u]*. Then, on each strong edge geodesic cover of H, v lies.*

**Proof.** Assume that *S* is the geodesic strong edge cover of *H* and v∈V. We must demonstrate that v∈S. Let v∉S if at all possible. Given that u∈N(v), *u*,*v* are adjacent. The edge (*u*,*v*) must lie on a strong geodesic *P* uniting some pair of nodes, say *a*,*b* of *S*, as *S* is a strong edge geodesic cover of *H*. Undoubtedly, v≠a,b. Let *t* be an additional node that is next to *v* and distinct from *u*. We observe that *u*,*t* are also adjacent as N(v)⊆N[u], which runs counter to the idea that *P* is a strong geodesic of *H*. As so, *v* lies on each geodesic cover of *H* with a strong edge.

**Theorem 14.** *If* H=(V,σ,μ) *be an mPFG having no δ-strong arcs where* |V|=n*. Then gn*_*e*_(*H*) = *n if and only if for each node a of H,* N(a)⊆N[b] *for some* b∈N(a).

**Proof.** Assume that *gn*_*e*_(*H*) = *n*. We’ll use a paradoxical approach to demonstrate this point. Let any node d∈N(c), if at all feasible, have a node *c* of *H* such that N(c)⊆N[d]. Assume S=V−{c}. We attempt to establish that *S* is a geodesic cover of *H* with a strong edge.

Let an edge of *H* be (*p*,*q*). p,q∈S if c∉{p,q}. Consequently, *S* encompasses the edge (*p*,*q*).

**Case 1:** Let *c* = *q*. Then p∈N(c). By the given hypothesis we have N(c)⊈N[p]. Therefore, there exists a node *t* in *N*(*c*) such that *t* is not in *N*[*p*]. Clearly, t≠p. Therefore the edge (*p*,*q*) lies on *p*–*q*–*t*. Thus *S* is a strong edge geodesic cover of *H*. Therefore, $gn_{e}(H)\leq |S|$, a contradiction.

**Case 2:** Let *p* = *c*. By continuing as above, we can demonstrate that *S* is a geodesic cover with a strong edge for *H*. Consequently, there is a contradiction $gn_{e}(H)\leq |S|$.

By combining everything said above, we obtain N(a)⊆N[b] for each node *a* of *H* for some b∈N(a).

On the other hand, consider that N(a)⊆N[b] for every node *a* of *H*, given that b∈N(a). Next, we have *c* sits on each strong edge geodesic cover of *H*, derived from Theorem 13. Consequently, *gn*_*e*_(*H*) = *n*.

## 5 Application

A key mathematical tool for representing real-world occurrences that are connected by graphical networks, in which nodes and edges represent *m*-polar fuzzy information, is the *m*PFG. Here, we apply the notion of a strong edge geodesic number inside a *m*PFG to a particular kind of covering problem.

### 5.1 Model construction

**Fig 5:**
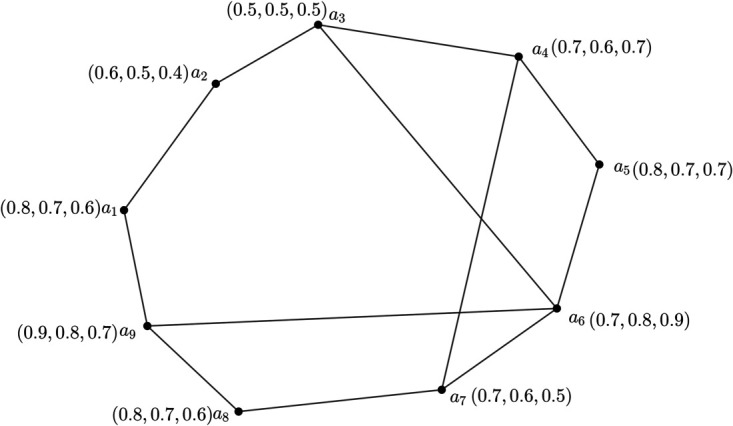
Model 3PFG *G* for bus route network system of some road network.

These days, a nation’s economic progress is greatly influenced by its transportation system. Well-organized planning will help to prevent loss and ensure that traffic moves smoothly. As a result, the covering problem is the core issue with the current research topic. Path covering is one of the more significant issues among them. Here, we talk about a path covering issue that has strong edge geodesic properties. Here, we study a road network represented as a *m*PFG, in which every node is a bus depot and an edge connects two nodes if bus routes exist between these depots. Our goal is to ascertain the bare minimum of traffic inspectors required to supervise and examine the bus routes on the network of metropolitan roads. At the same time, we want to find routes that passengers find less important so that they can be eliminated and reduce the losses that transportation companies suffer from inadequate collection. One may wonder if we can assign the MV of nodes and edges when we use 3PFG to represent this road network. Given that it has nine nodes and is a 3PFG. As a result, three factors will be needed to determine the maximum value of nodes, and three unique variables all of which must be uncertain?will be needed to display the maximum value of edges. Three factors will be taken into account while calculating the nodes MV: {the depot’s capacity, location, and services rendered}. The efficiency of bus route transportation is significantly influenced by the depot’s capacity, location, and services rendered. The depot serves as the operational hub where buses are maintained, refueled, and dispatched, making its capacity a crucial determinant of fleet size and service frequency. A strategically located depot minimizes deadhead miles distance traveled without passengers thereby reducing fuel costs, time wastage, and environmental impact. Additionally, the range of services rendered, such as maintenance, cleaning, and driver facilities, ensures operational reliability, vehicle longevity, and improved service quality. Collectively, these factors directly impact route efficiency, resource utilization, and passenger satisfaction. Optimizing depot characteristics is therefore fundamental for sustainable and cost-effective bus transportation systems. Each of the three factors varies depending on the depot and is characterized by uncertainty. Three factors will be taken into account when calculating the edges MV: {the state of the bus route, the signal delay, and the satisfied number of passengers}. The 3PFG model is presented in Fig 5.

Here, the edges membership value is given in tabular form in Table 5 for better visualization of the model 3PFG *G*.

**Table 5:** Edge membership value of the proposed model.

Edge	Membership Value
$\left(a_{1},a_{2}\right)$	(0.5,0.4,0.3)
$\left(a_{2},a_{3}\right)$	(0.5,0.4,0.3)
$\left(a_{3},a_{4}\right)$	(0.5,0.4,0.3)
$\left(a_{4},a_{5}\right)$	(0.7,0.6,0.5)
$\left(a_{5},a_{6}\right)$	(0.6,0.5,0.4)
$\left(a_{6},a_{7}\right)$	(0.6,0.5,0.4)
$\left(a_{7},a_{8}\right)$	(0.5,0.4,0.3)
$\left(a_{8},a_{9}\right)$	(0.7,0.6,0.5)
$\left(a_{1},a_{9}\right)$	(0.7,0.6,0.5)
$\left(a_{6},a_{9}\right)$	(0.4,0.3,0.2)
$\left(a_{3},a_{6}\right)$	(0.3,0.2,0.1)
$\left(a_{4},a_{7}\right)$	(0.1,0.2,0.3)

### 5.2 Illustration of membership values

The connectivity between the town nodes in the model network system which consists of nine nodes and twelve edges?is investigated about a specific 3PFG. First, the emphasis is on identifying if the relationships between the towns are *α*-strong, *β*-strong, or *δ*-strong.

We determine which edges are *α*-strong, *β*-strong, or *δ*-strong by calculating *CONN*_*G*−(*a*,*b*)_ for all (a,b)∈E, where *E* is the collection of edges in the model 3PFG. Then, using conventional calculations, the edges are categorized appropriately. For reference, the final edge categorization is shown in tabular form in Table 6.

**Table 6:** Classification of edges given in tabular form.

Edge	Classification
$\left(a_{1},a_{2}\right)$	*β*-strong
$\left(a_{2},a_{3}\right)$	*β*-strong
$\left(a_{3},a_{4}\right)$	*β*-strong
$\left(a_{4},a_{5}\right)$	*α*-strong
$\left(a_{5},a_{6}\right)$	*α*-strong
$\left(a_{6},a_{7}\right)$	*α*-strong
$\left(a_{7},a_{8}\right)$	*β*-strong
$\left(a_{8},a_{9}\right)$	*α*-strong
$\left(a_{1},a_{9}\right)$	*α*-strong
$\left(a_{6},a_{9}\right)$	*δ*-strong
$\left(a_{3},a_{6}\right)$	*δ*-strong
$\left(a_{4},a_{7}\right)$	*δ*-strong

**Fig 6:**
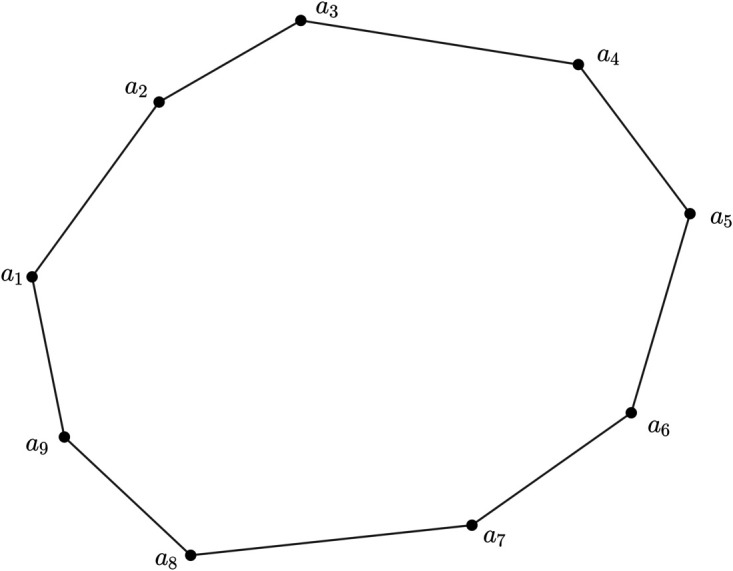
New 3PFG *G*_1_ corresponding to the Fig 5.

Here, we see that some arcs are *δ*-strong. From Theorem 4, we say that if a 3PFG has a strong edge geodesic number then it must not have a *δ*-strong arcs. Hence, we delete the *δ*-strong arcs from Fig 5 and we get the new 3PFG which is shown in Fig 6, where the existing nodes as well as edges have the same MV as of *G* described in the Fig 5. Since, we have to find out the solution of the problem stated above we must have an *m*PFG which has no *δ*-strong arcs and for our model *δ*-strong arcs are less priority route or the routes where minimum number of passengers will travel through bus. So, we delete all *δ*-strong arcs in the Fig 5 to convert the Fig 6.

### 5.3 Decision making

If we consider a set $U=\{a_{1},a_{5},a_{6}\}$, then we see that (U)=E, for the 3PFG *G*_1_. It is easy to check that *U* has the least number of elements among all the subsets of *V* whose geodesic cover is *E*. Therefore, the strong edge geodesic number of *G*_1_ is 3. Here, we also see that *U* is not unique. One may consider $U_{1}=\{a_{2},a_{6},a_{7}\}$. Then it is obvious to see that *U* as well as *U*_1_ have the same properties. One may find any other set having cardinality 3. Hence, instead of twelve traffic inspectors only three traffic inspectors are necessarily required to oversee and inspect the bus routes within the urban road network. Additionally, after assessing passenger preferences, it is evident that routes such as $\left(a_{3},a_{6}\right)$, $\left(a_{6},a_{9}\right)$, and $\left(a_{4},a_{7}\right)$ are receiving lower priority as *δ*-strong arcs are less priority route or the routes where minimum number of passengers will travel through bus. Therefore, to minimize losses resulting from insufficient revenue collection across various transport corporations, the elimination of these routes is proposed.

## 6 Advantages, disadvantages and limitations of the proposed work

In real-life scenarios, many problems are tackled using data collected from diverse sources, leading to multi-polarity. Such data, encompassing various origins, poses challenges for traditional fuzzy models, intuitionistic fuzzy models, or bipolar fuzzy models. For instance,we study a road network represented as a *m*PFG, in which every node is a bus depot and an edge connects two nodes if bus routes exist between these depots. Our goal is to ascertain the bare minimum of traffic inspectors required to supervise and examine the bus routes on the network of metropolitan roads. At the same time, we want to find routes that passengers find less important so that they can be eliminated and reduce the losses that transportation companies suffer from inadequate collection. Three factors will be taken into account while calculating the nodes MV: {the depot’s capacity, location, and services rendered}. The 3-polar fuzzy model is employed. Unlike traditional fuzzy models that handle single-component concepts, and bipolar or intuitionistic FG models limited to two components per edge or node, the *m*PFG model accommodates three components for each node and edge, offering more efficient fuzziness results. The development and analysis of such *m*PFGs, along with associated examples and theorems, enhance existing concepts and provide reliable solutions for complex real-world problems.

Additional advantages of the proposed model include:

(i) The ability for anyone to analyze MVs within a multi-polar fuzzy environment in a specific manner.
(ii) Presentation of a real application of ascertain the bare minimum of traffic inspectors required to supervise and examine the bus routes on the network of metropolitan roads. The *m*PF model utilizing the concept of geodesic.
(iii) Exploration of the concepts of multi-polarity in geodesic cover, basis within the *m*PFG environment.

The proposed model also has some disadvantages:

(i) It cannot accommodate negative MVs for the characters within this environment.
(ii) Non-heterogeneous types of data cannot be utilized within this framework.

Here are some limitations of this study:

(i) The *m*PFIG cannot be effectively applied if the MVs of the characters are provided in different interval-valued *m*PF environments.
(ii) The suggested methodology’s wider applicability across several disciplines, like the m-polar fuzzy graph notion, is limited by its primary application to networking systems.

## 7 Comparative study

At first, Bhutani and Rosenfeld [10] introduced a strong arcs for fuzzy graph theory. Next, they studied geodesic distance for crisp graph [11]. All of them studied strong arcs as well as edge geodesic distance for crisp graph and fuzzy graph theory. Later on, Suvarna and Sunitha [35] first introduced the concept of geodesic number for fuzzy graph. This concept is applicable only for fuzzy graphs. Later on, Reshmani and Sunitha [34] proposed a model to design edge geodesic number for fuzzy graph concept. So, all the earlier results are not applicable when the model is considered in another environment like in *m*-polar fuzzy sets. This is why the proposed model in this paper plays a significant role in such situations to give better results.

## 8 Conclusions

This paper introduces and defines the concepts of geodesic numbers and strong edge geodesic numbers within the context of *m*PFGs. It investigates the strong edge geodesic number for several well-known *m*PFGs and establishes an upper bound for this parameter. Additionally, the paper explores relationships between strong geodesic and strong edge geodesic numbers, while also delving into intriguing properties of the latter. The concept of edge geodesic numbers plays a critical role in graph theory, particularly in determining the minimum number of edges that intersect all shortest paths (geodesics) within a graph. The concept of the edge geodesic number, which quantifies the minimum number of edges required to maintain geodesic connectivity in a graph, has gained increasing interest in classical and fuzzy graph theory. In recent years, the extension of this parameter to fuzzy and m-polar fuzzy graphs has opened new avenues for exploration due to their ability to model uncertainty and multi-polarity in complex systems. This abstract proposes future directions in the study of the edge geodesic number on m-polar fuzzy graphs, emphasizing the development of novel algorithms to compute this invariant under varying degrees of membership, non-membership, and hesitation. Potential applications in network resilience, data mining, and decision-making systems are discussed, where multi-polarity plays a critical role. Further, we outline the theoretical challenges in defining edge geodesic preservation under *m*-polar constraints, propose generalizations to weighted and directed m-polar fuzzy graphs, *m*-polar intuitionistic fuzzy graphs, *m*-polar interval-valued intuitionistic fuzzy graphs and suggest comparative studies with other fuzzy graph parameters. This direction of study promises to deepen our understanding of structural properties in fuzzy environments and provide robust tools for real-world modeling.

## References

[1] Akram M, Adeel A. m-Polar fuzzy graphs and m-polar fuzzy line graphs. J Discrete Math Sci Cryptogr. 2017;20(8):1597–617. doi: 10.1080/09720529.2015.1117221

[2] Akram M, Wassem N and Dudek W A. Certain types of edge m-polar fuzzy graph. Iran J Fuzzy Syst. 2016 14(4): 27–50.

[3] Akram M. m-Polar fuzzy graphs. Springer International Publishing; 2019. 10.1007/978-3-030-03751-2

[4] Akram M. Bipolar fuzzy graphs. Inform Sci. 2011;181(24):5548–64. doi: 10.1016/j.ins.2011.07.037

[5] Akram M, Shumaiza, Rodríguez Alcantud JC. Multi-criteria decision making methods with bipolar fuzzy sets. Springer Nature Singapore; 2023. 10.1007/978-981-99-0569-0

[6] Akram M, Musavarah S, Wieslaw D. Energy of bipolar fuzzy graphs. Singapore: Springer; 2020. 10.1007/978-981-15-8756-6-8

[7] Akram M, Saleem D, Ghorai G. Energy of m-polar fuzzy digraphs. Advances in computer and electrical engineering. IGI Global; 2020. p. 469–91. 10.4018/978-1-5225-9380-5.ch020

[8] Alcantud JCR, Stojanović N, Djurović L and Laković M. Decision-making and clustering algorithms based on the scored-energy of hesitant fuzzy soft sets. Int J Fuzzy Syst. 2025, 1–15.

[9] Atici M. On the edge geodetic number of a graph. Int J Comput Math. 2003;80(7):853–61. doi: 10.1080/0020716031000103376

[10] Bhutani KR, Rosenfeld A. Strong arcs in fuzzy graphs. Inform Sci. 2003;152:319–22. doi: 10.1016/s0020-0255(02)00411-5

[11] Bhutani KR, Rosenfeld A (2004) Geodesics in fuzzy graphs. Appl Math Computat. 150(1), 65–75.

[12] Chartrand G, Harary F, Zhang P. On the geodetic number of a graph. Networks. 2001;39(1):1–6. doi: 10.1002/net.10007

[13] Chartrand G, Harary F, Zhang P. Geodetic sets in graphs. Discuss Math Graph Theory. 2000;20:129–38.

[14] Chen J, Li S, Ma S, Wang X. m-Polar fuzzy sets: An extension of bipolar fuzzy sets. ScientificWorldJournal. 2014;2014:416530. doi: 10.1155/2014/416530 25025087 PMC4082898

[15] Ghorai G, Pal M. On some operations and density of m-polar fuzzy graphs. Pacific Sci Rev A: Nat Sci Eng. 2015;17(1):14–22. doi: 10.1016/j.psra.2015.12.001

[16] Harary F, Loukakis E, Tsouros C. The geodetic number of a graph. Math Comput Modell. 1993;17(11):89–95. doi: 10.1016/0895-7177(93)90259-2

[17] Lu C. The geodetic numbers of graphs and digraphs. Sci China Ser A. 2007;50(8):1163–72. doi: 10.1007/s11425-007-0048-x

[18] Kauffman A. Introduction a la theorie des sous-emsembles flous. Mansson et Cie; 1973.

[19] Linda JP, Sunitha MS. Geodesic and Detour distances in graphs and fuzzy graphs. Scholars’ Press; 2015.

[20] Mahapatra T, Pal M. Fuzzy colouring of m-polar fuzzy graph and its application. J Intell Fuzzy Syst. 2018;35(6):6379–91. doi: 10.3233/jifs-181262

[21] Mahapatra T, Ghorai G, Pal M. Fuzzy fractional coloring of fuzzy graph with its application. J Ambient Intell Human Comput. 2020;11(11):5771–84. doi: 10.1007/s12652-020-01953-9

[22] Alanazi AM, Muhiuddin G, Mahapatra T, Bassfar Z, Pal M. Inverse graphs in m-polar fuzzy environments and their application in robotics manufacturing allocation problems with new techniques of resolvability. Symmetry. 2023;15(7):1387. doi: 10.3390/sym15071387

[23] Muhiuddin G, Mahapatra T, Pal M, Alshahrani O, Mahboob A. Integrity on m-polar fuzzy graphs and its application. Mathematics. 2023;11(6):1398. doi: 10.3390/math11061398

[24] Mandal S, Sahoo S, Ghorai G and Pal M. Different types of arcs in m-polar fuzzy graphs with application. J Mult Valued Logic Soft Comput. 2018;34:263–82.

[25] Mandal S, Sahoo S, Ghorai G, Pal M. Application of strong arcs in m-polar fuzzy graphs. Neural Process Lett. 2018;50(1):771–84. doi: 10.1007/s11063-018-9934-1

[26] Mathew S, Sunitha MS. Fuzzy graphs: Basics, concepts and applications. Lap Lambert Academic Publishing; 2012.

[27] Mordeson JN, Nair PS. Fuzzy graph and fuzzy hypergraphs. Physica-Verlag Heidelberg; 2000.

[28] Pal M, Samanta S, Ghorai G. Modern trends in fuzzy graph theory. Springer; 2020.

[29] Mondal U, Mahapatra T, Xin Q, Pal M. Solution of road network problem with the help of m-polar fuzzy graph using isometric and antipodal concept. Sci Rep. 2023;13(1):6452. doi: 10.1038/s41598-023-33071-9 37081040 PMC10119182

[30] Mondal U, Mahapatra T, Xin Q, Pal M. Generalized m-polar fuzzy planar graph and its application. IEEE Access. 2023;11:138399–413. doi: 10.1109/access.2023.3339220

[31] Nair PS, Cheng S-C. Cliques and fuzzy cliques in fuzzy graphs. In: Proceedings joint 9th IFSA world congress and 20th NAFIPS international conference (Cat. No. 01TH8569). 2277–80. 10.1109/nafips.2001.944426

[32] Pal M, Samanta S, Ghorai G. Modern trends in fuzzy graph theory. Springer; 2020.

[33] Rosenfeld A. Fuzzy graphs, fuzzy sets and their application. New York: Academic Press; 1975. p. 77–95.

[34] Rehmani S and Sunitha SM. Perfect geodesic fuzzy graphs. Int J Pure Appl Math. 2018;120(6):973–81.

[35] Suvarna NT, Sunitha MS. Convexity and types of arcs and nodes in fuzzy graphs. Scholar’s Press; 2015.

[36] Sunitha SM, Mathew S. Fuzzy graph theory: A survey. Ann Pure Appl Math. 2013;4:92–110.

[37] Stojanović N, Laković M, Djurović L. Decision-making algorithm based on the energy of interval-valued hesitant fuzzy soft sets. Neural Comput Applic. 2025;37(16):9821–41. doi: 10.1007/s00521-025-11107-7

[38] Zadeh LA. Fuzzy sets. Information and control. 1965;8(3):338–53. doi: 10.1016/s0019-9958(65)90241-x

